# Ceftolozane/Tazobactam for the Treatment of Adults With Cystic Fibrosis: Results From a French Prospective Cohort Study

**DOI:** 10.1093/ofid/ofae391

**Published:** 2024-08-06

**Authors:** Pierre-Régis Burgel, Xavier Bourge, Carole Mackosso, Francois Parquin

**Affiliations:** Respiratory Medicine and National Cystic Fibrosis Reference Center, Hôpital Cochin, Assistance Publique–Hôpitaux de Paris, Paris, France; Université Paris Cité and Institut Cochin, Inserm Paris, France; ERN-LUNG cystic fibrosis Core Network, Frankfurt, Germany; MSD France, Puteaux, France; MSD France, Puteaux, France; Unité de Soins Intensifs Respiratoires Groupe de Transplantation Pulmonaire, Hôpital Foch, Paris, France

**Keywords:** carbapenem, cystic fibrosis, gram-negative bacteria, multidrug resistance, real-world evidence

## Abstract

**Background:**

People with cystic fibrosis (pwCF) are particularly susceptible to respiratory infections, including those caused by multidrug-resistant (MDR) pathogens. Ceftolozane/tazobactam (C/T) is an antibacterial agent combination active against MDR gram-negative bacteria that has shown promising results in isolates from pwCF. This subanalysis is the first extensive observation of real-world C/T use in pwCF.

**Methods:**

The multicenter observational CONDUCT study included consecutive patients, some with cystic fibrosis, who received ≥1 dose of C/T at 28 centers throughout France. Patients were treated according to hospital standards and followed up until the end of C/T treatment (EOT).

**Results:**

Among 260 patients who had received ≥1 dose of C/T, 63 were pwCF, including 12 with previous lung transplant. The median age was 34 years and 55.6% of patients were female. *Pseudomonas aeruginosa* was the most frequently isolated pathogen (n = 40/41 [97.6%]). Most tested *P aeruginosa* strains (n = 65/73 [91.5%]) and all other isolated strains (*Escherichia coli*, *Citrobacter koseri*, *Proteus mirabilis*, and *Serratia marcescens*) were susceptible to C/T. Most patients completed the treatment duration, including those with historical β-lactam hypersensitivity. Reasons for stopping treatment were planned EOT and improvement in condition; overall, 88.9% of patients (n = 56/63) experienced improvement in condition. No new safety signals were identified. Mean forced expiratory volume in 1 second improved from 1.33 L to 1.47 L before and after C/T treatment, respectively (n = 52; *P* = .057).

**Conclusions:**

C/T treatment was well tolerated and effective in pwCF, including those with previous β-lactam hypersensitivity.

## Teaser

Real-world analysis in France demonstrated the effectiveness and tolerability of ceftolozane/tazobactam in people with cystic fibrosis, including lung transplant recipients and those with a history of hypersensitivity reaction to other β-lactam antibiotics.

Cystic fibrosis (CF) is estimated to affect at least 100 000 people globally [1]. In France, there are approximately 7700 people with CF (pwCF), 1340 of whom received inpatient treatment in 2022 [2]. CF is a multisystem genetic disease caused by mutations in the gene encoding the CF transmembrane conductance regulator (CFTR) protein. These mutations can result in the abnormal transport of chloride and bicarbonate ions across epithelial surfaces. Disrupted ion transport depletes airway surface liquid and impedes mucociliary clearance, resulting in airway infection and inflammation, bronchiectasis, and impaired lung function [1, 3–5]. CF eventually leads to respiratory failure and is one of the most common reasons for lung transplantation; up to 20% of adults with CF currently live with lung transplant [1].

*Pseudomonas aeruginosa* is a gram-negative bacterium found in the airways of 50%–75% of adults with CF, and many patients are chronically infected [6, 7]. Infection with *P aeruginosa* can result in acute pulmonary exacerbations, often leading to increased inflammation, structural damage to the lungs, lung function decline, and premature mortality in pwCF [7, 8]. *Pseudomonas aeruginosa* can be isolated from the lower airways of pwCF after lung transplantation and may be responsible for lower respiratory tract infections, such as bronchitis and pneumonia [9, 10].

PwCF often initially acquire a strain of *P aeruginosa* with an in vitro antibiotic-susceptible phenotype, which can be cleared with aggressive treatment. However, *P aeruginosa* can persist due to resistant cells, resulting in a chronic infection that cannot be cleared with antibiotics [11].

Repeated exposure to antimicrobial agents increases the risk of antibiotic resistance or patient hypersensitivity to antibacterial agents [12, 13]. Consequently, some strains of *P aeruginosa* found in the airways of pwCF have become progressively resistant to multiple antibiotics. In the United States (US) in 2020, 3.5% of pwCF with ≥1 bacterial culture had multidrug-resistant (MDR) *P aeruginosa* [14]. New antimicrobial agents are urgently needed to address increasing antibiotic resistance in pwCF [15].

A challenge associated with the development of antimicrobial agents is that many studies of antibiotic treatment in pwCF show a dissociation between clinical effectiveness and in vitro susceptibility [16]. Therefore, there is agreement among CF experts that antibiotic regimen choice in pwCF should be guided by bacterial species identification and previous antibiotic treatment, rather than predominantly relying on in vitro susceptibility testing [17].

Ceftolozane/tazobactam (C/T; ZERBAXA, Merck Sharp & Dohme LLC, a subsidiary of Merck & Co, Inc, Rahway, New Jersey) is a cephalosporin paired with a β-lactamase inhibitor. C/T is active against extended-spectrum β-lactamase (ESBL)–producing Enterobacteriaceae and MDR *P aeruginosa* [18]. C/T received marketing authorization in the European Union (EU) in 2015 and was initially approved in the US in 2014 [19, 20]. C/T is indicated for the treatment of complicated intra-abdominal infection, complicated urinary tract infection, and acute pyelonephritis in adult and pediatric patients in the US and EU. C/T is also indicated for the treatment of hospital-acquired bacterial pneumonia (HABP), including ventilator-associated bacterial pneumonia (VABP), in adult patients in the US and EU [18, 19, 21]. Healthcare professionals have described case reports of positive outcomes for patients with CF treated with C/T [22–25]; however, there are limited data on the use of C/T in pwCF.

Conditions of Post marketing Use of Ceftolozane/Tazobactam (ZERBAXA) in Real-World Settings (CONDUCT) was an observational, postmarketing study requested by the Transparency Commission of the French National Authority of Health to describe the demographic and clinical characteristics, and real-world treatment patterns, among hospitalized patients treated with C/T [26]. In October 2020, the CONDUCT protocol was modified to assess specific characteristics of patients with CF treated with C/T, due to the unexpected observation that they represented a large proportion of those treated with C/T in France. This article presents the results of the CONDUCT subanalysis in patients with CF. It is the first of its kind to explore the real-world use of C/T in pwCF, including those living with a lung transplant.

## METHODS

This analysis reports observations from the CONDUCT study, specifically in patients with CF. The primary objective was to describe the demographic and clinical characteristics, in addition to the real-world treatment patterns and outcomes, among hospitalized patients with CF treated with C/T.

### Study Duration

The recruitment period was 14 months (first patient: 11 October 2018; last patient: 30 December 2019). The study duration, including the observational period, was 17 months (11 October 2018 to 21 March 2020). Patients were followed up until C/T cessation (last dose received).

### Participating Center Selection

The study setting was limited to hospitals in France. Twenty-eight centers proficient in the diagnosis and management of gram-negative bacterial infections were selected to participate. The following data were collected as part of a feasibility study during center solicitation and selection: type of hospital (hospital center, university hospital center, or clinic); prescription services and number of prescribing physicians; presence of an antibiotics therapy advisor; type of local microbiology laboratory (full-service facility or exclusive service); availability of on-site resistance testing; and availability of bacterial storage system (cryogenic vials, −80°C freezer, or centrifuges).

### Patient Selection

Patients with CF who had received at least 1 dose of C/T and consented to participate were eligible for inclusion in the present analysis. Each patient was treated according to each center's routine practice. The decision to treat was at the physicians' discretion and was made prior to each patient's inclusion in the study.

Diagnosis of CF was confirmed using validated international criteria [27]. Microbiology results were collected from available antibiograms and medical records from participating centers.

Data collected from patients included history of lung transplantation; previous intravenous antibiotic therapy for lung infection (within the past 3 years); sensitivity to β-lactam antibiotics; context of C/T prescription; spirometry results (forced expiratory volume in 1 second [FEV_1_], if available at C/T initiation and cessation); last available bacterial culture results in the context of lung infection; and susceptibility to C/T, if antibiograms were available. Antibiograms were not available for all patients, as susceptibility testing is not always carried out for samples from pwCF, owing to high cost and low clinical utility [28]. The number of patients with immunosuppression was also recorded; immunosuppression was defined as the long-term use of corticosteroids or other immunosuppressive drugs (eg, anticalcineurin or mammalian target of rapamycin inhibitors).

### Treatment Dosing

Patients were treated with C/T at a dose decided by their treating physician based on their infection status and recommended dosage [18]. C/T received market authorization for pneumonia (HABP and VABP) on 23 August 2019; therefore, C/T doses given to patients with pneumonia included after this date followed the updated summary of product characteristics guidelines [18]. At the time of the study, pwCF were not treated with elexacaftor–tezacaftor–ivacaftor, which became available for clinical use (outside of clinical trials) in France in December 2019.

### Bacteriological Analyses

Initial bacteriological analyses of sputum samples, to identify bacterial strains, determine minimum inhibitory concentration (MIC), and quantify colonies, were performed at local laboratories according to local protocols. Following local laboratory testing, duplicate samples were stored at −80°C using the MAST CRYOBANK (Mast Group Ltd, United Kingdom) system. One duplicate per case was sent to a central laboratory for final analyses. The central laboratory tested bacterial isolates for MIC cut-offs.

Bacterial isolates demonstrating antibiotic resistance were tested for the presence of inhibitory compounds (ie, ESBLs and carbapenemases) using molecular methods (polymerase chain reaction and sequencing). Isolates that could not be identified, or that presented rare or unknown forms of resistance, were sent to a national reference laboratory for further analysis.

### Statistical Analyses

All statistical analyses were performed using SAS software (version 9.4 or later; SAS Institute, Cary, North Carolina). This observational study was descriptive and aimed to investigate the real-life prescription and use of C/T. Patients living with a lung transplant were compared with nontransplanted patients. Paired Wilcoxon test was performed to compare FEV_1_ before and after treatment.

### Patient Consent Statement

This study was approved by the French ethics committee, Comité de Protection des Personnes, in accordance with the French Data Protection Authority (Commission Nationale de l’Informatique et des Libertés), and conforms to local ethical standards. Patients who agreed to participate in the study provided written informed consent.

## RESULTS

### CONDUCT Study

CONDUCT was a multicenter study investigating the real-world clinical outcomes and use of C/T. A total of 260 patients who had received ≥1 dose of C/T were recruited from 28 specialist centers across France (Supplementary Figure 1).

### CONDUCT Subanalysis in Patients With CF

#### Patient Characteristics

In total, 24.2% (n = 63/260) of patients in the CONDUCT study were living with CF (Table 1), of whom 19.0% (n = 12/63) had received previous lung transplantation. The mean age of pwCF was 33.6 years (standard deviation [SD], 9.0; range, 13.7–59.4) and most pwCF were female (55.6%). Most pwCF (60.3%) had normal weight (body mass index [BMI], 18.5–25.0 kg/m^2^), but 34.9% of patients had low (<18.5 kg/m^2^) BMI and 4.8% of patients were overweight (BMI 25.0–30.0 kg/m^2^; Table 1). These findings were not unexpected, as low BMI and malnutrition are common in pwCF [29].

**Table 1.** Baseline Characteristics of Patients With Cystic Fibrosis, Including Those Living With Lung Transplant, Treated With Ceftolozane/Tazobactam

Characteristic	Patients Without Transplantation (n = 51)	Patients With Transplantation (n = 12)	Total pwCF (n = 63)	*P* Value
Age, y				.290
n	51	12	63	
Mean (SD)	34.2 (9.2)	31.1 (8.1)	33.6 (9.0)	
Median	34.2	33.7	34.0	
Q1–Q3	28.4–38.5	24.5–38.3	27.1–38.3	
Range	13.7–59.4	18.4–41.7	13.7–59.4	
Sex				.132
n	51	12	63	
Male	25 (49.0)	3 (25.0)	28 (44.4)	
Female	26 (51.0)	9 (75.0)	35 (55.6)	
BMI category				.035
n	51	12	63	
Underweight (<18.5 kg/m^2^)	14 (27.5)	8 (66.7)	22 (34.9)	
Normal (18.5–24.9 kg/m^2^)	34 (66.7)	4 (33.3)	38 (60.3)	
Overweight (≥25.0–30.0 kg/m^2^)	3 (5.9)	0	3 (4.8)	
Obese (>30.0 kg/m^2^)	0	0	0	
Comorbidities				.190
n	51	12	63	
No	28 (54.9)	6 (50.0)	34 (54.0)	
Yes	23 (45.1)	6 (50.0)	29 (46.0)	
CF-related diabetes	18 (35.3)	4 (33.3)	22 (34.9)	1.000
Moderate or severe hepatic disease	3 (5.9)	0	3 (4.8)	1.000
Renal failure	2 (3.9)	4 (33.3)	6 (9.5)	.010
Other	5 (9.8)	0	5 (7.9)	.573
Creatinine clearance				.058
n	51	12	63	
Missing data	10 (19.6)	1 (8.3)	11 (17.5)	
>150 mL/min (glomerular hyperfiltration)	2 (3.9)	0	2 (3.2)	
50–150 mL/min	37 (72.5)	7 (58.3)	44 (69.8)	
30–49.9 mL/min	0	1 (8.3)	1 (1.6)	
15–29.9 mL/min	1 (2.0)	1 (8.3)	2 (3.2)	
<15 mL/min (severe renal impairment)	1 (2.0)	2 (16.7)	3 (4.8)	
Patients with immunodepression				<.001
n	51	12	63	
No	44 (86.3)	0	44 (69.8)	
Yes	7 (13.7)	12 (100.0)	19 (30.2)	
FEV_1_				.987
FEV_1_ measured before C/T initiation				
n	47	10	57	
Mean (SD)	1.3 (0.6)	1.3 (0.6)	1.3 (0.6)	
Median	1.2	1.2	1.2	
Q1–Q3	0.9–1.5	0.8–1.8	0.9–1.5	
Range	0.5–4.1	0.5–2.3	0.5–4.1	
Prior IV antibacterial therapy in the 2 y prior to C/T initiation				
n	50^a^	12	62	
Patients with 1 antibacterial treatment	4 (8.0)	2 (16.7)	6 (9.7)	
Patients with ≥2 antibacterial treatments	46 (92.0)	10 (83.3)	56 (90.3)	
Hypersensitivity drug reaction to antibiotics^b^				
Patients with hypersensitivity to β-lactams				
n	51	12	63	
Yes	29 (56.9)	3 (25.0)	32 (50.8)	.047
Known history of hypersensitivity to antibacterial agents	29	3	32	1.000
Hypersensitivity to 1 antibacterial agent	6 (20.7)	0	6 (18.8)	
Hypersensitivity to 2 antibacterial agents	5 (17.2)	0	5 (15.6)	
Hypersensitivity to ≥3 antibacterial agents	18 (62.1)	3 (100.0)	21 (65.6)	
Hypersensitivity to β-lactam antibiotics^b^	29	3	32	.047
Carbapenems^b^	19 (65.5)	3 (100.0)	22 (68.8)	.534
Imipenem	3 (10.3)	1 (33.3)	4 (12.5)	
Meropenem	17 (58.6)	3 (100.0)	20 (62.5)	
Cephalosporins^b^	25 (86.2)	0	25 (78.1)	.007
Ceftazidime	25 (86.2)	0	25 (78.1)	
Cefuroxime	1 (3.4)	0	1 (3.1)	
Combinations with β-lactamase inhibitor^b^	11 (37.9)	3 (100.0)	14 (43.8)	.073
Ceftazidime/avibactam	0	2 (66.7)	2 (6.3)	
Amoxicillin/clavulanic acid	1 (3.4)	0	1 (3.1)	
Piperacillin/tazobactam	10 (34.5)	2 (66.7)	12 (37.5)	
Monobactam (aztreonam)	12 (41.4)	0	12 (37.5)	.274
Penicillins	4 (13.8)	0	4 (12.5)	1.000
Penicillin	2 (6.9)	0	2 (6.3)	
Ticarcillin	2 (6.9)	0	2 (6.3)	

Data are presented as No. (%) unless otherwise indicated.

Abbreviations: BMI, body mass index; CF, cystic fibrosis; C/T, ceftolozane/tazobactam; FEV_1_, forced expiratory volume in 1 second; IV, intravenous; pwCF, people with cystic fibrosis; Q, quartile; SD, standard deviation.

^a^Data were missing for 1 patient.

^b^Patient may have allergies/intolerances to ≥2 antibiotics at the same time.

All pwCF included in this analysis had chronic lung disease and 46.0% (n = 29/63) had additional medical conditions, including CF-related diabetes (34.9%) and renal failure (9.5%; Table 1). Renal failure was more common in pwCF who had undergone lung transplant than in those who had not (33.3% [n = 4/12] vs 3.9% [n = 2/51]; *P* = .010). Rates of CF-related diabetes or moderate or severe hepatic disease were comparable between patients with and without lung transplant (Table 1). The vast majority of pwCF (90.3% [n = 56/62], data missing for 1 patient) had received 2 or more intravenous antibacterial therapies in the 2 years prior to starting C/T therapy (Table 1; Supplementary Figure 2).

Approximately half of patients (50.8%) had a history of a hypersensitivity reaction to at least 1 β-lactam antibiotic; of these patients, 78.1% were allergic to ceftazidime and 62.5% to meropenem (Table 1).

#### Infection Characteristics

Overall, 96.8% of patients (n = 61) had pulmonary exacerbation and/or pneumonia and 3.2% (n = 2) had pneumonia with bacteremia. Most patients started treatment with C/T in a pneumology department (73% [n = 46/63]) versus 11.1% (n = 7/63) in intensive care units and 15.9% in other hospital departments (Supplementary Table 1).

#### Microbiology

*Pseudomonas aeruginosa* was the most common pathogen isolated at local laboratories (100% of samples [n = 36]; Table 2) and at the central laboratory from sputum samples that underwent centralized strain analysis (97.6% of samples [n = 40/41]; Table 3). *Pseudomonas aeruginosa* comprised 89.9% (n = 71/79) of all strains isolated at the central laboratory (Table 3). Antibiograms performed immediately before onset of treatment were available for 28 patients, 46.4% (n = 13/28) of which had been tested by the central laboratory. C/T-susceptible strains were identified in 89.3% (n = 25/28) of these patients, of whom a high percentage had antibiograms reporting resistance to cefepime (78.6%), ceftazidime (82.1%), and piperacillin/tazobactam (78.6%).

**Table 2.** Microbiology Results, Including Susceptibility and Resistance Profile to Ceftolozane/Tazobactam in the Local Laboratory

Bacteria Identified	No. of Patients (n = 36)	No. of Strains (n = 91)	Susceptible to C/T (n = 74)	Resistant to C/T (n = 17)
*Pseudomonas aeruginosa*	36 (100)	89 (97.8)	72 (80.9)	17 (19.1)
*Escherichia coli*	1 (2.8)	1 (1.1)	1 (100)	0 (0)
*Serratia marcescens*	1 (2.8)	1 (1.1)	1 (100)	0 (0)

Data are presented as No. (%). Strains for which there was a local antibiogram were not all sent to the central laboratory to be tested. Bacterial species coming from the same laboratory may have been tested several times.

Abbreviation: C/T, ceftolozane/tazobactam.

**Table 3.** Microbiology Results and Susceptibility of Isolates to Ceftolozane/Tazobactam in the Central Laboratory

Bacteria Identified	No. of Patients^a^ (n = 41)	No. of Strains (n = 79)	Strains Susceptible to C/T (n = 73)	Strains Resistant to C/T (n = 6^b^)
*Pseudomonas aeruginosa*	40 (97.6)	71 (89.9)	65 (91.5)	6 (8.5)
*Escherichia coli*	3 (7.3)	4 (5.06)	4 (100)	0 (0)
*Citrobacter koseri*	1 (2.4)	2 (2.5)	2 (100)	0 (0)
*Proteus mirabilis*	1 (2.4)	1 (1.3)	1 (100)	0 (0)
*Serratia marcescens*	1 (2.4)	1 (1.3)	1 (100)	0 (0)

Data are presented as No. (%).

Abbreviation: C/T, ceftolozane/tazobactam.

^a^One documented patient and 3 patients with empirical treatment had >1 strain (*P aeruginosa* + *E coli* + *S marcescens*; *P aeruginosa* + *E coli*; *P aeruginosa* + *C koseri*; *E coli* + *P mirabilis*).

^b^All 6 resistant strains were isolated from patients with empirical prescription.

Strains of *P aeruginosa* isolated in the central laboratory demonstrated high rates of susceptibility to C/T (91.5%; Supplementary Table 4). While 85.2% of *P aeruginosa* strains isolated in local laboratories were susceptible to C/T, less than 26% of isolates were susceptible to the other antibiotics (Table 3). All other identified isolates were 100% susceptible to C/T (Table 3); these isolates were *Escherichia coli*, *Citrobacter koseri*, *Proteus mirabilis*, and *Serratia marcescens*.

#### Treatment Effectiveness and Safety

The median treatment duration in this study was 15 days, which is consistent with the recommended treatment length in pwCF, and most pwCF completed full treatment. At the end of C/T treatment (EOT), 88.9% (n = 56/63) of patients experienced improvement in condition (Table 4). Empiric treatment for suspected infection in pwCF, administered according to hospital protocols, was the most common type of antibacterial therapy (77.8%). Most patients had a history of resistance or a previous antibiogram available on file. Patients who received empiric prescriptions could have their treatment adapted after receiving microbiology results. In pwCF, a previous result is often used to guide the choice of antimicrobial agent(s) prescribed [17]. Most patients (69.8% [n = 44/63]) were prescribed concomitant antimicrobial agents that were initiated either before (22.7% [n = 10/63]), or at the same time as (77.3% [n = 34/63]), treatment with C/T (Supplementary Table 2; Supplementary Table 3).

**Table 4.** Reasons for Stopping Treatment

Reason for Stopping Treatment	No. (%) of Patients (N = 63)
Improvement in condition	56 (88.9)
Adaptation to microbiology results	1 (1.6)
Occurrence of an adverse event^a^	2 (3.2)
Treatment failure	1 (1.6)
Other reason for stopping	3 (4.8)
Planned discontinuation of antibiotic therapy	1 (33.3)
Unable to continue in day care hospital for financial reasons^b^	1 (33.3)
External medical decision	1 (33.3)

^a^One patient reported pruritus and another patient had a skin rash that was possibly related to a hypersensitivity reaction.

^b^Ceftolozane/tazobactam (C/T) could be prescribed exclusively in hospital. The patient received 10 days of treatment with C/T in total.

**Figure 1.**
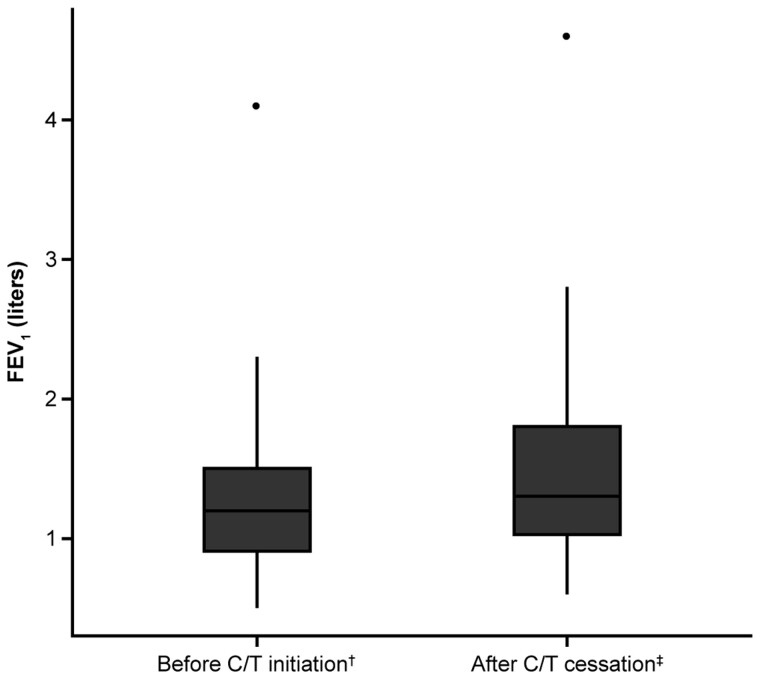
Comparison of forced expiratory volume in 1 second (FEV_1_) in 52 people with cystic fibrosis with measurement taken at ceftolozane/tazobactam (C/T) treatment initiation and after cessation. The center line denotes the mean FEV_1_ value while the box contains the standard deviation values. ^†^FEV_1_ before initiation of treatment was defined as an FEV_1_ measurement taken within the 6-month period leading up to C/T treatment initiation, or at the time of C/T treatment initiation, or 14 days before the end of treatment. ^‡^FEV_1_ after cessation of treatment was defined as an FEV_1_ measurement taken when treatment with C/T was completed. FEV_1_ measurements taken before cessation of treatment that were considered as “after treatment cessation” were all those performed if the participant had completed treatment.

Mean FEV_1_ increased after treatment with C/T (1.33 L [SD, 0.6; range, 0.50–4.10] vs 1.47 L [SD, 0.7; range, 0.60–4.60] before and after treatment, respectively; n = 52; *P* = .057; Figure 1).

A total of 3.2% of patients (n = 2) experienced an adverse event that resulted in treatment discontinuation (Table 4). No new safety concerns related to C/T were identified, despite some patients being prescribed treatment doses that were higher than typically recommended.

## DISCUSSION

This subanalysis of a real-world observational study (CONDUCT) found that treatment with C/T was effective and well tolerated in pwCF in France. In the CONDUCT study, approximately 25% of C/T prescriptions were for pwCF; this large number of C/T prescriptions is remarkable and is high relative to the number of pwCF in France, who represent approximately 0.01% of the French population.

The most common bacterial isolate was *P aeruginosa*, which was identified in 97.6% of patients (n = 40/41). Most *P aeruginosa* isolates (91.5%), and all other bacterial species isolated from patients with CF, were susceptible to C/T; however, only 22%–26% of strains were susceptible to 3 other tested β-lactam antibiotics. β-lactam–resistant bacteria are common in pwCF; therefore, these data indicate that C/T is an effective tool for treating infections in this patient population.

A trend toward improved FEV_1_ was observed following treatment with C/T, suggesting that C/T treatment may contribute to improved respiratory function in this patient population. However, longitudinal studies are required to assess the impact of C/T on lung function in pwCF. These studies will help to develop statistical models to approach nonlinear changes in lung function, quantify variability, and assess the influence of risk factors [30, 31].

pwCF have high exposure to antibiotics, which can lead to high rates of antimicrobial-resistant infections and the development of allergic reactions [12, 32]. In this study, although half of patients reported hypersensitivity to parenteral β-lactam antibiotics, most completed the treatment until an improvement in condition was observed. C/T showed a good safety profile; adverse events were reported for only 2 of the 63 (3.2%) patients, and no additional treatment was required.

C/T has a unique molecular profile, providing a treatment option that is well tolerated and results in low rates of allergic reaction. Furthermore, C/T has a comparable safety profile to other antibiotics, has low rates of discontinuation, and is suitable for treating MDR infections. Importantly, these results were achieved in a population who had received large numbers of intravenous antibiotic courses in the years prior to the trial and had high rates of hypersensitivity to β-lactams.

CFTR modulators are highly effective and reduce the need for intravenous antibiotics; however, they are not suitable for all patients and do not clear bacterial infection, so do not completely suppress the need for intravenous antibiotics [33–35]. Therefore, effective antimicrobial agents for pwCF are still required and C/T represents a suitable treatment option for this patient population. Patients in the present study were not treated with the highly effective combination therapy elexacaftor–tezacaftor–ivacaftor, owing to a lack of availability at the time of this study [36]. Therefore, there is scope for further investigation into the safety and efficacy of concomitant elexacaftor–tezacaftor–ivacaftor and C/T therapy.

A strength of this study is the selection of specialist centers for treating CF, all belonging to the French CF Reference network; patients were treated by experts who understood their treatment needs and provided a high standard of care. Furthermore, bias related to patient selection was limited by the inclusion of consecutive patients. This subanalysis is the first real-world evidence study to focus on the use of C/T in pwCF, which is important, as the high percentage of patients treated with C/T was not anticipated during approval by the European Medicines Agency. These results provide evidence to support a new indication of C/T to treat respiratory infections in pwCF, including after lung transplantation.

The main limitation of the study was its observational nature. As C/T is only available for prescription in a hospital setting, the patients in the study were generally critically ill and were thus closely followed in an intensive care unit in several cases. In addition, not all patients had samples taken at treatment initiation or had their samples analyzed at the centralized laboratory, which reflects the practice of antibiotic prescription to pwCF based on previous results of microbiological analysis of sputum samples and previous antibiotic use [17]. Furthermore, the study was performed before elexacaftor–tezacaftor–ivacaftor therapy was introduced (see above). However, CFTR modulators are seldom used posttransplantation, and 10%–20% of pwCF are ineligible for this triple-combination therapy owing to their genotype [37]. An additional limitation was that not all strains were available for sending to the central laboratory, which likely explains most of the differences in the percentage of C/T-resistant strains between local laboratories (19.1% [n = 17/89 strains]) and the central laboratory (8.1% [n = 6/79]). Part of this difference could also be related to the use of conventional techniques used at the local laboratory versus the central laboratory.

C/T has been shown to be effective in the context of outpatient parenteral antibiotic therapy (OPAT), including to successfully treat a patient with CF who was infected with MDR *P aeruginosa* and ESBL-producing *E coli* [38]. The success of C/T in the OPAT setting is partly due to its stability of up to 24 hours at room or body temperature, in accordance with EU and US standards, and up to 12 hours in alignment with United Kingdom standards [39–41]. The observed stability and clinical success of C/T in OPAT indicate that C/T is useful for the treatment of MDR infections in inpatient and outpatient settings. OPAT using C/T could provide benefits to patients, including cost savings, improved quality of life, and reduced hospital stays and risk of nosocomial infections [39]. The results of this subanalysis support the clinical success of C/T; there is scope for further studies evaluating C/T for outpatient and at-home treatment of pwCF.

## CONCLUSIONS

Overall, results from this subanalysis suggest that treatment with C/T is effective in pwCF, including those with MDR infections and those living with a lung transplant. Although pwCF are not mentioned explicitly in the product label for C/T, the results from this study and several clinical observations suggest that C/T could prove to be a well-tolerated, useful resource in treating infections, and could address an unmet need in this patient population.

## Supplementary Data

Supplementary materials are available at *Open Forum Infectious Diseases* online. Consisting of data provided by the authors to benefit the reader, the posted materials are not copyedited and are the sole responsibility of the authors, so questions or comments should be addressed to the corresponding author.

## Supplementary Material

ofae391_Supplementary_Data

## Notes

***Acknowledgments.*** The authors thank the participants, their families, and all investigators involved in this study. Medical writing support was provided by Sophie Byrne, MSci, and Lauren Moreton, MRes, and editorial support was provided by Ian Norton, PhD, all of Scion (a division of Prime, London, UK), supported by Merck Sharp & Dohme LLC, a subsidiary of Merck & Co, Inc, Rahway, New Jersey, USA, according to Good Publication Practice guidelines.

***Author contributions.*** All authors designed and conceived the study, acquired and analyzed data, interpreted results, and drafted and reviewed the manuscript. All authors provided final approval of the version to be published. All authors agree to be accountable for all aspects of the work in ensuring that questions related to the accuracy or integrity of any part of the work are appropriately investigated and resolved.

***Data availability.*** The data sharing policy, including restrictions, of Merck Sharp & Dohme LLC, a subsidiary of Merck & Co, Inc, Rahway, New Jersey, USA, is available at http://engagezone.msd.com/ds_documentation.php. Requests for access to the clinical study data can be submitted through the EngageZone site or via email to the Data Access mailbox.

***Financial support*.** This study was funded by Merck Sharp & Dohme LLC, a subsidiary of Merck & Co, Inc, Rahway, New Jersey, USA.
