# Supplementary material for: Ceftolozane/Tazobactam for the Treatment of Adults With Cystic Fibrosis: Results From a French Prospective Cohort Study
Source: Open Forum Infect Dis. 2024 Aug 6;11(8):ofae391. doi: 10.1093/ofid/ofae391 (PMC11303002; doi:10.1093/ofid/ofae391)
Supplement: ofae391_Supplementary_Data [file ofae391_supplementary_data.docx]

# Supplementary Material

Supplementary Table 1. Description of Department Initiation of C/T for pwCF

| **Department, n (%)** | **pwCF (N = 63)** |
| --- | --- |
| Pneumology | 46 (73.0) |
| ICU | 7 (11.1) |
| Thoracic and cardiovascular surgery | 2 (3.2) |
| Other | 8 (12.7) |

Abbreviations: C/T, ceftolozane/tazobactam; ICU, intensive care unit; pwCF, people with cystic fibrosis.

Supplementary Table 2. Concomitant Antibiotic Treatments Administered to pwCF

|  | **Documented^a^ (n = 14)** | **Empiric^b^ (n = 49)** | **Total (N = 63)** |
| --- | --- | --- | --- |
| **Patients prescribed concomitant antibiotics, n (%)** | 8 (57.1) | 36 (73.5) | 44 (69.8) |
| **Number of concomitant antibiotics per patient, n (%)** |  |  |  |
| N | 8 | 36 | 44 |
| 1 | 6 (75.0) | 27 (75.0) | 33 (7.0) |
| 2 | 2 (25.0) | 8 (22.2) | 10 (22.7) |
| 3 | 0 | 1 (2.8) | 1 (2.3) |
| Antibiotic initiated before C/T | 3 (37.5) | 7 (19.4) | 10 (22.7) |
| Antibiotic initiated at the same time as C/T | 5 (62.5) | 29 (80.6) | 34 (77.3) |

Abbreviations: C/T, ceftolozane/tazobactam; pwCF, people with cystic fibrosis.
^a^Prescription according to antibiogram results.
^b^Prescription before the antibiogram results were received.

Supplementary Table 3. Description of Concomitant^a^ Treatment for Index Infection by Antibiotic Class

| **Concomitant antibiotics administered, n (%)** | **Patients receiving documented C/T prescription (n = 8)** | **Patients receiving empiric C/T prescription (n = 36)** | **Total (n = 44)** |
| --- | --- | --- | --- |
| **One concomitant antibiotic** | 6 | 27 | 33 |
| Aminoglycosides | 5 (83.3) | 17 (63.0) | 22 (66.7) |
| Fluoroquinolones | 0 (0.0) | 8 (29.6) | 8 (24.2) |
| Oxazolidinones | 0 (0.0) | 1 (3.7) | 1 (3.0) |
| Other | 1 (16.7) | 1 (3.7) | 2 (6.1) |
| N | 6 | 26 | 32 |
| Mean duration of concomitant treatment, days (SD) | 9.3 (4.6) | 13.5 (5.4) | 12.7 (5.4) |
| Median | 7.5 | 15.0 | 15.0 |
| Range | 4.0–15.0 | 4.0–24.0 | 4.0–24.0 |
| **Two concomitant antibiotics** | 2 | 8 | 10 |
| Aminoglycosides + oxazolidinones | 0 (0.0) | 1 (12.5) | 1 (10.0) |
| Fluoroquinolones + other | 0 (0.0) | 1 (12.5) | 1 (10.0) |
| Fluoroquinolones + oxazolidinones | 0 (0.0) | 1 (12.5) | 1 (10.0) |
| Glycopeptides and lipoglycopeptides + aminoglycosides | 0 (0.0) | 1 (12.5) | 1 (10.0) |
| Macrolides, lincosamides + aminoglycosides | 0 (0.0) | 1 (12.5) | 1 (10.0) |
| Aminoglycosides + other | 1 (50.0) | 0 (0.0) | 1 (10.0) |
| Tetracyclines + aminoglycosides | 1 (50.0) | 3 (37.5) | 4 (40.0) |
| N | 3 | 11 | 14 |
| Mean (SD) | 9.3 (6.5) | 17.3 (6.0) | 15.6 (6.8) |
| Median | 9.0 | 16.0 | 15.5 |
| Range | 3.0–16.0 | 4.0–26.0 | 3.0–26.0 |
| **Three concomitant antibiotics** | – | 1 | – |
| Glycopeptides and lipoglycopeptides + macrolides, lincosamides + tetracyclines | – | 1 (100.0) | – |

Abbreviations: C/T, ceftolozane/tazobactam; SD, standard deviation.
^a^Concomitant antibiotics (i.e., antibiotics administered for at least 2 days along with C/T).

Supplementary Table 4. Susceptibility and Resistance Profile of *Pseudomonas aeruginosa* to Cefepime, Ceftazidime, Piperacillin/Tazobactam, and C/T based on Antibiograms for 28 pwCF from Local Laboratories

| **Microbiology results, n (%)^a,b^** | **Cefepime (N = 97)** | **Ceftazidime (N = 99)** | **Piperacillin/tazobactam (N = 98)** | **C/T (N = 101)** |
| --- | --- | --- | --- | --- |
| Susceptible | 25 (25.8) | 22 (22.2) | 27 (27.6) | 82 (81.2) |
| Intermediate | 0 | 0 | 1 (1.0) | 0 |
| Resistant | 72 (74.2) | 77 (77.8) | 70 (71.4) | 19 (18.8) |

Abbreviations: C/T, ceftolozane/tazobactam; pwCF, people with cystic fibrosis.
^a^Based on antibiograms for 28 patients.

^b^Some of the 79 strains isolated were tested more than once.

Supplementary Figure 1. Selection of PwCF


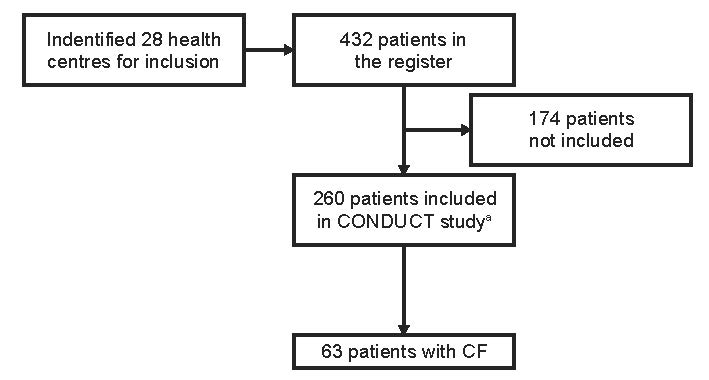


^‡^

^†^

Abbreviations: CF, cystic fibrosis; pwCF, people with cystic fibrosis.

^†^The protocol aimed to include 30 centers; however, 28 were ultimately included.

^‡^Two included patients not reported in the register.

## Supplementary Figure 2. Classes of Previous Antibacterial Agents Prescribed for the Treatment of the Index Infection Prior to C/T Initiation, for Patients in the Overall CONDUCT Study (N = 260)

^‡^**^†^**

^†^

Abbreviations: C/T, ceftolozane/tazobactam. ^†^Prescription according to antibiogram results. ^‡^Prescription before the antibiogram results were received.
